# Effect of Transradial Artery Catheterization on Shock Patients

**DOI:** 10.1155/2022/8746066

**Published:** 2022-09-28

**Authors:** Hui Xu, Wenyong Chen, Mingming Huang, Zenggeng Wang

**Affiliations:** ^1^Department of Emergency, Jiangxi Provincial People's Hospital, Nanchang, Jiangxi 330006, China; ^2^Department of Emergency, Yongxiu County People's Hospital, Jiujiang, Jiangxi 330300, China; ^3^Department of Emergency, Gongqingcheng People's Hospital, Jiujiang, Jiangxi 332020, China

## Abstract

**Objective:**

This study aimed to investigate the clinical effect of ultrasound-guided transradial catheterization (TRC) for ICU patients with shock.

**Methods:**

120 shock patients registered in the ICU of our hospital from January 2019 to June 2022 were selected for prospective study. The control group (60 patients) were treated with palpation-guided TRC. The observation group was treated with ultrasound-guided TRC and was divided into the internal puncture group (internal TRC) and external puncture group (external TRC), with 30 cases in each. The first attempt success rate, total success rate, operation duration, complication, measurement of radial artery, and VAS scores were compared in these groups.

**Results:**

The success rate was higher in the observation group than in the control group (*P* < 0.05), and higher in the internal puncture group than in the external puncture group (*P* < 0.05). The first attempt success rate was significantly higher in the observation group than in the control group (*P* < 0.05), with no significant difference in between (*P* > 0.05). The number of attempts and operation duration were lower in the observation group than in the control group (*P* < 0.05), with significantly more operation duration in the internal puncture group than in the external puncture group (*P* < 0.05) and no significant difference in the number of attempts (both *P* > 0.05). The complication rate was significantly lower in the observation group than in the control group (*P* < 0.05) and there was no significant difference in between (*P* > 0.05). The radial artery diameter, cross-sectional area, and depth from the skin in the observation group were larger than those in the control group (*P* < 0.05) and there was no significant difference in between (*P* > 0.05). At 1, 6, 24, and 48 h after the surgery, the observation group showed lower VAS scores than the control group (*P* < 0.05).

**Conclusion:**

The ultrasound-guided TRC reduced the number of attempts, the complication rates, and the operation duration. For patients with shock, if Doppler ultrasound cannot detect blood flow, the success rate in the observation group was higher than that in the control group, and its advantage is worthy of promotion in severe patients.

## 1. Introduction

The term “shock” refers to a life-threatening circulatory failure caused by an imbalance between the supply and demand of cellular oxygen [1, 2]. The condition of shock patients changes rapidly, and the hemodynamic is extremely unstable. After hemodynamic disorder, intracoronary blood flow decreases, resulting in insufficient myocardial blood supply and oxygen supply [3]. Blood pressure and mean arterial pressure are two of the most important outcome measures in diagnosis and treatment for shock. It has become a routine treatment for shock patients to establish a vascular access with both real-time monitoring of blood pressure and convenient arterial blood collection as soon as possible. Meanwhile, with the surgical interventions for cardiovascular disease being taken widely, transradial catheterization (TRC) has gradually become one of the first options as the operative approach for interventional surgery due to the unaffected postoperative activities, quick postoperative hemostasis by compression, and easier observation of postoperative bleeding.

The radial artery is usually the first choice for arterial catheterization, because the radial and ulnar arteries have a rich lateral circulation that avoids ischemic necrosis of the anterior limb. However, obesity, ectopia, hypoperfusion (hypotension, low cardiac output), extreme-weak arterial beats, and arterial spasm can all lead to failed arterial catheterization [4, 5]. In recent years, ultrasound guidance has been widely used in jugular catheterization, peripherally inserted central venous catheterization, and femoral venous catheterization, but there have been few applications reported in artery catheterization. For instance, ultrasound guidance for epidural surgery has gained popularity and interest, especially for lumbar epidural needle placement and catheterization, and its application in thoracic epidural surgeries has also been receiving attention [6]. Ultrasound-guided catheterization, as a “visual” new technology [7], makes the radial artery imaging clear and directly observes the radial artery orientation, anatomical variants, and the neighboring relationship with the surrounding tissues, which helps to find the best puncture point, improve the success rate, and reduce the injury with catheterization [8].

This study aimed to investigate the clinical value of ultrasound-guided TRC for ICU patients with shock, which may provide a novel insight for clinical application in severe patients.

## 2. Clinical Materials and Methods

### 2.1. General Clinical Data

120 shock patients registered in the ICU of our hospital from January 2019 to June 2022 were selected for prospective study. Inclusion criteria: Patients met the diagnostic criteria for shock [9]. Exclusion criteria: Patients with a history of forearm surgery, local infection, local arterial embolization, or abnormal results of the quantitative SaO2-Allen test (negative). A total of 120 patients were included, aged from 15 to 95 years old, with a mean age of 62.13 ± and 19.81 years old. Patients enrolled in the study were randomized into the control and observation groups using a computer-generated list in the ratio of 1 : 1. The control group was treated with palpation-guided TRC (blind method). The observation group treated with ultrasound-guided TRC was divided into the internal puncture group (internal TRC) and the external puncture group (external TRC). There were 60 cases in the control group and 30 cases in either the internal puncture group or external puncture group. In the control group, there were 31 males and 29 females, aged 15.0–81.0 (64.3 ± 3.5), with a PACHEII score of 36.8 ± 5.3. In the external puncture group, there were 16 males and 14 females, aged 21.0–80.0 (65.1 ± 3.3), with a PACHEII score of 35.2 ± 4.6. In the internal puncture group, there were 17 males and 13 females, aged 20–95 (64.6 ± 3.1), with a PACHEII score of 36.5 ± 4.9. In addition, prior to TRC, the purpose and process of TRC were explained to all the patients themselves or their family members, and the informed consent form was signed by the authorized family members to obtain understanding and cooperation.

### 2.2. Methods

Patients were taken in the supine position or lifted 30 degrees by their heads, routinely assessed for radial artery beating, and evaluated with the Allen test. In both the groups, artery catheterization was processed using a 22G catheter from B. Braun.

The control group was treated with palpation-guided TRC. After disinfection of the left index and middle fingers, the operator palpated the artery beat using the left index and middle fingers to determine the puncture site. The catheter held by the operator's right hand was punctured into the radial artery along the arterial direction at 15°–30° oblique angle no matter using the direct method or the penetrating method and set lower when flashbacks of arterial blood were observed. The needle was withdrawn when the radial arterial blood flow was pressed and blocked by the end of the catheter near the heart. Then the cannula was connected to the disposable blood pressure transducer, disinfected, and fixed.

The authorized medical staff of our hospital (the first author of this paper) performed ultrasound-guided TRC in the observation group using a unified ultrasound system (M5s/M7 Series portable color Doppler ultrasound system, Shenzhen Mindray Biomedical Electronics Co., LTD.) with 7.5∼10 MHz line array probe (7L4s). Patients were placed in the supine position or lifted 30 degrees by their heads and 15 degrees with their feet, with the left or right upper limb stretched out at 30°–60° and the wrist back stretched out at about 30° to fully expose the radial artery. A nursing cushion was placed underneath. Before the operation, the vessel condition and puncture site were determined according to the inclusion and exclusion criteria, and the relevant diameters were recorded. Internal puncture group: the disposable medical sterile protective cover was used for ultrasound probe protection. The probe was filled with couplant and fixed. The color Doppler system was set to upper limb artery mode. The vascular pulse and target puncture site were determined by color Doppler flow imaging (CDFI). The puncture was processed using the internal method. The pinpoint of the catheter was adjusted under color Doppler localization till it entered into the vessel. The catheter was withdrawn for fixation when the flashback of blood was observed. External puncture group: the disposable medical sterile protective cover was used for ultrasound probe protection. The probe was filled with couplant and fixed. The color Doppler system was set to upper limb artery mode. The vascular pulse and target puncture site were determined by CDFI. The puncture was processed using the external method. After the pinpoint was punctured into the center of the vascular lumen, the cannula was pushed forward by the left hand and the needle was drawn back by the right hand. If putting forward was not smooth, the direct method was switched to the penetrating method to withdraw the needle and put forward the cannula when the beating blood spouted. After successful puncture, a large disposable transparent film was used for fixation and the disposable pressure transducer was connected. All operations were performed by the same operator. On account of the severe shock of patients, another doctor was responsible for the emergency treatment and emergency operation during the operation.

### 2.3. Outcome Measures

1. *First Attempt Success Rate*. The proportion of patients with successful first attempt of TRC to the total patients in group. First attempt success rate = successful first attempt cases/total cases *∗* 100%.
2. *Total Success Rate*. The proportion of patients with successful TRC to the total patients in group. Total success rate = total successful cases/total cases *∗* 100%.
3. *Number of Attempts*. Number of attempts required for successful TRC.
4. *Complication Rate*. During the catheterization, the proportion of patients with complications to the total patients (complications criteria: ① Local hematoma: swelling, ecchymosis, bulge, and waving feeling in the local skin near the catheter; ② puncture site bleeding: After successful TRC, blood flowing out from the puncture site; ③ local infection: Local redness and swelling near the catheter. Complication rate = complication cases/total cases *∗* 100%.
5. *Operation Duration*. Time spent for successful TRC in both the groups. The control group recorded the time spent from palpation to successful TRC. The observation group recorded the time spent for ultrasound-guided TRC, including the image localization time (time from the probe being placed on the skin to the needle being punctured into the skin) and the catheterization duration.
6. *Vascular Diameter, Cross-Sectional Area, and Depth from the Skin*. Measurement of the parameters were performed using the Sonosite built-in software.
7. *Pain Status at 1, 6, 24, and 48 h after Surgery*. The visual analogue scale (VAS) score was used to assess pain degree, with a total score of 10, higher score indicating more severe pain.

### 2.4. Statistical Analysis

We planned to enroll 120 patients. We calculated that with this sample size the study would have 90% power to detect an increase in the first attempt success rate or total success rate from 70% in the control group to 90% in the puncture group at an *α* level of .05. The internal puncture and external puncture groups belonged to the observation group. They were estimated and both have an effect on shock patients. Thus, we used a separation ratio of 2 : 1 : 1.

Statistical analysis was conducted with SAS version 9.3 (SAS Institute Inc). All statistical tests were 2-sided. *P* < 0.05 was considered statistically significant.

## 3. Results

### 3.1. Comparison of General Data

The patients' age, sex, acute physiology, and acute Physiology and chronic health evaluation (PACHEII) were compared, and the results showed no significant difference (all *P* < 0.05), suggesting they were comparable.

### 3.2. Comparison of the Puncture Conditions

**Figure 1:**
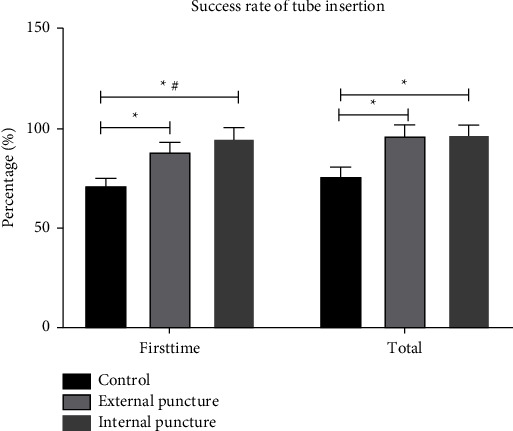
The puncture conditions in three groups. ^*∗*^*P* < 0.05, compared with the control group; ^#^*P* < 0.05, compared with the external puncture group.

The results showed that the total success rate of TRC was significantly higher in the observation group than in the control group (*P* < 0.05) and higher in the internal puncture group than in the external puncture group, with significant differences (*P* < 0.05). The first attempt success rate was significantly higher in the observation group than in the control group (*P* < 0.05), with no significant difference in between (*P* > 0.05), as shown in Figure 1.

### 3.3. Comparison of Attempts and Operation Duration

**Figure 2:**
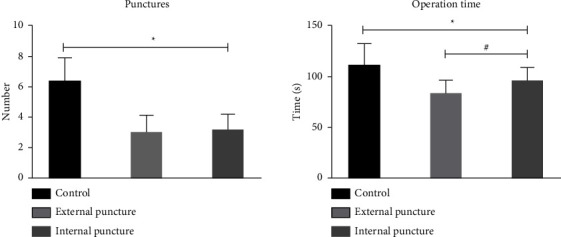
Attempts and operation duration in three groups. ^*∗*^*P* < 0.05, compared with the control group; ^#^*P* < 0.05, compared with the external puncture group.

The number of attempts and operation duration were less in the observation group than in the control group (*P* < 0.05), with significantly more operation duration in the internal puncture group than in the external puncture group (*P* < 0.05) and no significant difference in the number of attempts (both *P* > 0.05), as shown in Figure 2.

### 3.4. Comparison of Complications of Peripheral Arterial Catheters

The complication rate was significantly lower in the observation group than in the control group (*P* < 0.05) and there was no significant difference in between (*P* > 0.05), as shown in Table 1.

### 3.5. Comparison of Arterial Measurement and TRC-Related Information

**Figure 3:**
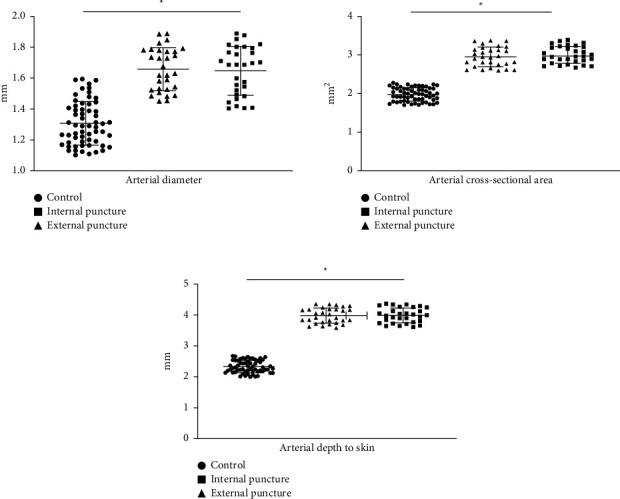
Arterial measurement and TRC-related information in three groups. ^*∗*^*P* < 0.05, compared with the control group.

The radial artery diameter, cross-sectional area, and depth from the skin in the observation group were larger than those in the control group (*P* < 0.05) and there was no difference in between (*P* > 0.05), as shown in Figure 3.

### 3.6. Comparison of Postoperative Pain Status

**Figure 4:**
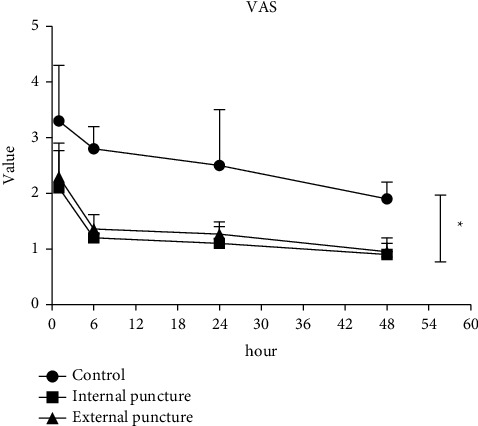
Postoperative pain status in three groups ^*∗*^*P* < 0.05, compared with the control group.

At 1, 6, 24, and 48 h after the surgery, the observation group showed lower VAS scores than the control group (*P* < 0.05) and there was no significant difference between the external puncture group and the internal puncture group (*P* > 0.05), as shown in Figure 4.

## 4. Discussion

The condition of shock patients changes rapidly with the possibility of massive bleeding, causing the patients to undergo frequent arterial blood collection and invasive arterial blood pressure monitoring. Radial artery blood collection has the advantages of small pain response, easy acceptance by patients, less pressure and preparation time, and less occurrence of hematoma, so the radial artery has become the preferred route of arterial catheterization for invasive blood pressure monitoring [10–12]. In clinical practice, however, there are often some shock patients in critical condition who have weak artery pulses due to limb swelling, insufficient capacity, and poor circulation. The traditional blind catheterization consists of anatomical localization and the palpation of the radial artery, which has a low first attempt success rate and causes multiple attempts at puncture, making it stressful and painful for patients awake. It is also easy to cause radial artery spasm, bleeding, hematoma, and other complications, leading to puncture failure or catheterization and blood collection difficulty [13, 14]. The ultrasound-guided TRC clearly shows the arterial blood vessels and their surrounding tissues, and by using the high-quality imaging of the blood vessels by the ultrasound, the thickness, depth, and walking of the radial artery are clearly seen under the ultrasound. An external puncture is used during the puncture, and the radial artery image is placed in the middle of the probe and kept fixed. The needle is punctured through the midpoint of the probe, which can greatly improve the success rate of arterial catheterization and reduce or even avoid the related complications [15–17].

Ultrasound technology has been widely used in clinical vascular puncture and has become the third “eye” of medical staff, reducing related complications and thus becoming more and more popularized in clinical practice. In recent years, with the emergence of high-frequency and high-definition ultrasound equipment and the continuous improvement of ultrasound probes, the application of ultrasound-guided TRC in the rescue and interventional treatment of acute and critical diseases is also on the rise, with more and more related studies. At present, ultrasound-guided catheterization includes internal puncture and external puncture with each having their own advantages and disadvantages and is been widely used for regional nerve block and central venipuncture. When the long-axis internal puncture is used, the ultrasound probe, the sheath, and the long-axis of vessel are in the same plane, and the long-axis section of the vessel and the trocar can be fully displayed during the puncture process, but the position relationship between the pinpoint and the vessel in the short-axis section cannot be clearly displayed. On the other hand, the cross-sectional image of the vascular lumen captured during external puncture can clearly show the relationship between the positions of the trocar and the vessel in the short axis section, but sometimes it does not define the exact location of the pinpoint. Because the related complications of arterial puncture are more serious than those of venous puncture, how to reduce the complications is the focus of the current research. The results of this study showed that patients in need of TRC, palpation-guided group, may have multiple trials before success, which increased the number of attempts and operation duration. Compared to the palpation-guided control group, the ultrasound-guided group showed an increased first attempt success rate, significantly reduced number of attempts, less operation duration, and a lower VAS score in both the internal puncture group and the external puncture group, indicating that the ultrasound-guided TRC is a very effective and safe catheterization technique. It is worth noting that although the external puncture group was not significantly different from the internal puncture group in overall success rate, its first attempt success rate and operation duration were lower than those in the internal puncture group, and the reason for this difference may be related to the proficiency in the two methods. When using internal puncture, the left hand needs to fix the probe position continuously, and a little movement may lose the puncture plane. Therefore, internal puncture is more difficult than external one, and the beginners mostly prefer to use external puncture. Ball et al. [18] believe that although internal puncture has higher technical difficulty, it is clinically safer because the clear imaging of the needle tip in the lumen is better to avoid the vascular damage caused by catheter. When using external puncture, if the punctured blood vessel is relatively thin, due to the strong pressure of the anterior wall on the inner blood vessel caused by the large angle of insertion it can easily flatten the vessel and make the vessel lumen thinner, resulting in the vague position of the needle and a high risk of perforation through the posterior wall, thus causing hematoma. When internal puncture is used, its small angle of insertion causes limited pressure of the anterior wall to the blood vessel, with inconspicuous lumen changes. In addition, the position of the needle can be observed during the whole process, which can avoid the perforation through the posterior wall to a greater extent. Therefore, for shocked patients, the advantages of internal puncture are more prominent. Meanwhile, shock patients are often accompanied by coagulation dysfunction, so once the artery is perforated, it will easily cause hematoma and bleeding difficulties. Therefore, based on our research results, we suggested that internal puncture to be the main choice of TRC for shock patients.

Gu et al. [19] performed a meta-analysis of seven RCTs and showed that compared to palpation guidance, ultrasound guidance can improve the success rate of TRC and reduce the number of attempts, thus reducing the incidence of complications such as hematoma. In this study, the complication rate in the external puncture group and the internal puncture group was significantly lower than that in the control group. The patients involved were all critically ill, with insufficient volume, poor circulation, shock, systemic edema, and their arterial blood flow was unable to be seen using Doppler ultrasound. Therefore, the arterial catheterization became difficult, with inevitably repeated attempts, thus increasing the incidence of local hematoma, puncture bleeding, local infection, and so on. The external puncture group and the internal puncture group can quickly finish the localization of radial artery vessels and measure the size and depth of the arterial lumen. If the edema of the puncture site is too severe to form an image of blood flow, the arterial pulse of some patients can be observed alternately, thus solving the problem of arterial catheterization for severe patients and reducing the incidence of complications.

Although this study focused on patients with shock, the method we detected is suitable for every patient in need of TRC, and the success rate would be higher for patients with normal hemodynamics and good vascular filling. This method can also be used for patients undergoing radial puncture for interventional surgery. In addition, the operator's proficiency in ultrasound use is also key to the success of TRC. The operators of this study have all passed the standardized training on ultrasound for severe patients and have mastered the basic principles of ultrasound use, the selection and use of vascular probes, how to determine the artery/venous vessel and other ultrasound technologies. Due to the easy learning of ultrasound-guided puncture of blood vessels and its short training period, with the improvement of the hospital equipment, especially in the ICU wards, the equipment of ultrasound machines has been more and more popularized, creating better conditions for ultrasound-guided puncture. However, there were still some limitations. The sample size is quite small, thus we need more samples in our future research. Additionally, nonblinded design might have introduced bias, which should be avoided as much as possible in the future.

In conclusion, ultrasound-guided TRC made it possible to dynamically observe the radial artery vessels, thus benefiting by locating the puncture site and guiding the operation. It could effectively overcome the limitations of blind catheterization with simple, safe, and repeatable operations, greatly improving the first attempt success rate, reducing the number of attempts, reducing the incidence of complications, and reducing operation duration. Its superiority and practicability are worth promoting in severe patients.

## Figures and Tables

**Table 1:** Complications of peripheral arterial catheters in three groups.

Group	Complication			Total	Incidence rate (%)
	Local hematoma	Puncture site bleeding	Local infection		
Control	8 (13.33)	5 (8.33)	2 (3.33)	15	25.00
External puncture	1 (3.33)	3 (10.00)	0 (0.00)	4	13.33
Internal puncture	2 (6.67)	1 (3.33)	0 (0.00)	3	10.00

*t*	4.537				
*P*	<0.001				

## Data Availability

The analysed datasets generated during the study are available from the corresponding author on reasonable request.
